# Global Dynamics of the Stationary M_2_ Mode‐1 Internal Tide

**DOI:** 10.1029/2020GL091692

**Published:** 2021-06-05

**Authors:** Samuel M. Kelly, Amy F. Waterhouse, Anna C. Savage

**Affiliations:** ^1^ Large Lakes Observatory and Physics & Astronomy Department University of Minnesota Duluth Duluth MN USA; ^2^ Scripps Institution of Oceanography University of California La Jolla San Diego CA USA

**Keywords:** tide, internal tide, internal wave, physical oceanography

## Abstract

A reduced‐physics model is employed at 1/25° to 1/100° global resolution to determine (a) if linear dynamics can reproduce the observed low‐mode M_2_ internal tide, (b) internal‐tide sensitivity to bathymetry, stratification, surface tides, and dissipation parameterizations, and (c) the amount of power transferred to the nonstationary internal tide. The simulations predict 200 GW of mode‐1 internal‐tide generation, consistent with a general circulation model and semianalytical theory. Mode‐1 energy is sensitive to damping, but a simulation using parameterizations for wave drag and wave‐mean interaction predicts 84% of satellite observed sea‐surface height amplitude variance on a 1° × 1° grid. The simulation energy balance indicates that 16% of stationary mode‐1 energy is scattered to modes 2–4 and negligible energy propagates onto the shelves. The remaining 84% of energy is lost through parameterizations for high‐mode scattering over rough topography (54%) and wave‐mean interactions that transfer energy to the nonstationary internal tide (29%).

## Key Points

- Global mode‐1 internal tides are predictable from a linearized, reduced physics model
- The choice of damping parameterizations predetermines mode‐1 internal‐tide energy
- Simulations with damping by wave drag and wave‐mean interactions largely agree with satellite data

## 1 Introduction

Internal tides are internal waves at tidal frequencies that are generated where surface tides push stratified water up and down sloping topography (Garrett & Kunze, 2007). Globally, this process extracts 1.0 TW from all of the surface tides, with 0.8 TW from the M_2_ (12.42 h period) surface tide alone (Egbert & Ray, 2003). Some fraction (15%–50%) of internal‐tide energy is lost to turbulent dissipation near generation sites (e.g., Rudnick et al., 2003) and the remainder is radiated 1000s of km across the open ocean (Ray & Mitchum, 1996). As internal tides propagate through mesoscale turbulence they lose coherence with the surface tide (and astronomical tidal potential), becoming nonstationary (e.g., Rainville & Pinkel, 2006; Zaron, 2017; Nelson et al., 2019). Radiating internal tides also dissipate slowly through a combination of topographic scattering (e.g., Müller & Xu, 1992; Bühler & Holmes‐Cerfon, 2011; Mathur et al., 2014), wave‐meanflow interactions (e.g., Dunphy et al., 2017; Savage et al., 2020), and wave‐wave interactions (e.g., MacKinnon et al., 2013; Olbers et al., 2020). Although the relative role of each decay processes is unknown, the geography of internal‐tide dissipation (as it relates to diapycnal mixing) is a key ingredient in climate models (MacKinnon et al., 2017).

Accurate internal‐tide models are also critical for removing tidal contamination from satellite observations of mesoscale circulation (Carrere et al., 2021). The upcoming Surface Water Ocean Topography (SWOT) satellite mission will provide 2‐km resolution sea surface height (SSH) over a 100‐km wide swath. This data will contain mesoscale and internal tide SSH signals, but the internal tides cannot be removed using traditional time‐series analysis because the satellite has a 21‐days repeat orbit.

The stationary low‐mode M_2_ internal tide is the natural starting point for predicting internal tides, because it dominates global internal‐tide energy balances (e.g., Garrett & Kunze, 2007; Falahat et al., 2014; Buijsman, Stephenson, et al., 2020), is well observed by existing satellite altimeters (Carrere et al., 2021), and only requires statistical (not instantaneous) knowledge of small‐scale topographic scattering, wave‐wave interactions, and wave‐mean flow interactions (e.g., Savage et al., 2020). At present, global internal‐tide predictions are available via (a) empirical atlases based on fits to satellite data (Carrere et al., 2021), (b) semianalytical models of generation and propagation (de Lavergne et al., 2019), and (c) general circulation models with tidal forcing (Arbic et al., 2018). All three types of predictions are suspect in shallow coastal regions where stratification is highly variable and tides often have short wavelengths and large amplitudes. The first class of predictions are accurate for the stationary internal tide in the deep ocean, but difficult to extend to nonstationary internal tides (Carrere et al., 2021). The second class of predictions use the “weak topography” approximation for generation estimates (Bell, 1975) and the geometric approximation for ray tracing (Rainville & Pinkel, 2006). These tools have been used to estimate global energy balances (de Lavergne et al., 2019), but not predict internal‐tide amplitude and phase. The third class of predictions also explicitly calculate surface tides, stratification, and mesoscale variability, so these input/background fields deviate slightly from observations. General circulation models are also complicated, nonlinear, and computationally intensive, making them nontrivial to tune for optimal internal tides (Buijsman et al., 2016, 2020). Despite these challenges, general circulation models simulate the stationary low‐mode tide with reasonable skill (Kodaira et al., 2016; Zhao et al., 2016; Carrere et al., 2021).

Is there a prediction method that avoids the weak‐topography approximation and the complexity of general circulation models, while still retaining the leading‐order dynamics of the low‐mode internal tide? The Coupled‐mode Shallow Water model (CSW) fits this description (Kelly et al., 2016; Savage et al., 2020), but its dynamics have not yet been analyzed at a global scale. (Griffiths (2011) developed an analogous global model 10 years ago, but didn't report detailed results.) CSW describes linear, low‐mode internal tides as they interact with prescribed bathymetry, stratification, surface tides, and mesoscale flow. This model can include parameterizations of internal‐tide scattering by small scale roughness (Jayne & St. Laurent, 2001; Buijsman et al., 2016, 2020), loss of stationarity by wave‐mesoscale interaction (Savage et al., 2020), and/or generic unresolved processes, which Niwa and Hibiya (2014) represented through a spatially constant linear drag, $r_{\text{const.}}^{-1}=30$ days.

Although the CSW model resembles the “tides‐only” general circulation models of Simmons et al. (2004) and Niwa and Hibiya (2011), CSW is different because it (a) uses vertical modes rather than isopycnal or terrain‐following coordinates, (b) treats surface tides and stratification as input parameters, (c) includes updated decay parameterizations, and (d) is linear and, therefore, less computationally intensive to run and easier to reformulate as an inverse model. Rewriting CSW as an inverse model would essentially be a refinement of G. D. Egbert and S. Y. Erofeeva's internal‐tide inversion model (see Carrere et al., 2021), which represents the mode‐1 tide using a reduced‐gravity model.

Here we examine the dynamics and energetics of the stationary mode‐1 internal tide in CSW, a new type of global internal‐tide prediction model.

## 2 Methods

### 2.1 Equations of Motion

Equations describing the evolution of vertical mode amplitudes were first derived and applied by Griffiths and Grimshaw (2007) and Griffiths (2011). Here, we employ the CSW model used by Savage et al. (2020), which is asymptotically accurate for small‐amplitude internal tides in a small‐Rossby‐number (Ro ≪ 1) background flow. The model evolves the stationary vertical‐mode transports, **U**_*n*_(**x**, *t*), and pressures, *p*_*n*_(**x**, *t*), using

(1a) $$\begin{array}{ll} \frac{\partial U_{n}}{\partial t}+f\hat{k}\times U_{n} & =-H\nabla p_{n}-\overset{\infty }{\underset{m=0}{\sum }}HT_{mn}p_{m}-rU_{n}+\nu \nabla^{2}U_{n} \end{array}$$

(1b) $$\begin{array}{ll} \frac{H}{c_{n}^{2}}\frac{\partial p_{n}}{\partial t} & =-\nabla \cdot U_{n}+\overset{\infty }{\underset{m=0}{\sum }}T_{nm}\cdot U_{m}-r\frac{H}{c_{n}^{2}}p_{n}+\kappa \frac{H}{c_{n}^{2}}\nabla^{2}p_{n}, \end{array}$$

where *n* is the mode number, *f* the inertial frequency, *H*(**x**) the depth, and *c*_*n*_(**x**) the mode eigenspeed. The model includes both linear drag and viscosity/diffusivity terms to accommodate different parameterizations for damping, which are discussed in Section 2.2. Briefly, a small viscosity maintains numerical stability. Diffusivity is either zero or *κ*_mean_ (for mean flow effects). Linear drag is either zero, *r*_wave_ (for wave drag), or *r*_const._ (for generic unresolved processes). Topographic scattering matrices

(2) $$T_{mn}\left(x\right)=\frac{1}{H}\overset{0}{\underset{-H}{\int }}\phi_{n}\nabla \phi_{m}dz\;$$

couple vertical modes, *ϕ*_*n*_(*z*; **x**), which are orthogonal

(3) $$\frac{1}{H}\overset{0}{\underset{-H}{\int }}\phi_{m}\phi_{n}dz=\delta_{mn},$$

and determined, along with the eigenspeeds, by solving the Sturm‐Liouville problem

(4) $$\frac{d^{2}\Phi_{n}}{dz^{2}}+\frac{N^{2}}{c_{n}^{2}}\Phi_{n}=0\;with\;\Phi_{n}\left(0\right)=\Phi_{n}\left(-H\right)=0,$$

where *ϕ*_*n*_ = dΦ_*n*_/d*z*, and *N* ^2^ is the mean buoyancy frequency. The equations are forced by prescribing known surface‐tide transports, **U**_0_, in the topographic coupling terms.

The Steady‐state Mode‐*n* energy Balance is

(5) $$\nabla \cdot \underset{F_{n}}{\underset{︸}{\bar{U_{n}p_{n}}}}=\underset{C_{n}}{\underset{︸}{\bar{U_{0}p_{n}}\cdot T_{n0}}}-\underset{S_{n}}{\underset{︸}{\overset{\infty }{\underset{m=1}{\sum }}\left(\bar{U_{n}p_{m}}\cdot T_{mn}-\bar{U_{m}p_{n}}\cdot T_{nm}\right)}}\;-\underset{D_{n}}{\underset{︸}{\left(2rE_{n}-\nu \bar{\frac{U_{n}}{H}\cdot \nabla^{2}U_{n}}-\kappa \bar{\frac{Hp_{n}}{c_{n}^{2}}\nabla^{2}p_{n}}\right)}}\;,$$

where the overbar is a time average, and the terms are the divergence of energy flux (*F*_*n*_), generation (*C*_*n*_), topographic scattering (*S*_*n*_), and dissipation by drag and diffusion (*D*_*n*_). Total mode‐*n* energy is

(6) $$E_{n}=\frac{1}{2}\frac{\bar{|U_{n}{|}^{2}}}{H}+\frac{1}{2}\frac{H\bar{p_{n}^{2}}}{c_{n}^{2}}.$$

### 2.2 Numerical Implementation

The equations of motion (equation (1)) are discretized using a second‐order finite‐volume formulation on a spherical C‐grid and integrated in time using a second‐order Adams‐Bashforth algorithm. To eliminate grid‐scale noise and stabilize the model, we (a) limit the domain to locations deeper than 16 m, (b) only turn on the topographic‐coupling terms in locations deeper than 100 m, (c) limit surface‐tide forcing to locations deeper than 300 m, (d) ignore surface‐tide forcing in the weakly‐stratified Southern Ocean (south of 60° S, where *c*_1_ < 1 m s^1^ in some deep locations), and (e) include an $O\left(10\right)\;m^{2}\;s^{-1}$ numerical viscosity, *ν* (Bryan et al., 1975). The code is written in C with MPI to run in parallel on a supercomputer.

The vertical modes and eigenspeeds in equation (4) are found using a spectral method (Kelly, 2016) with global bathymetry and climatological stratification. Topographic coupling coefficients (equation (2)) are then computed at the center of each grid cell using centered differences.

**Figure 1:**
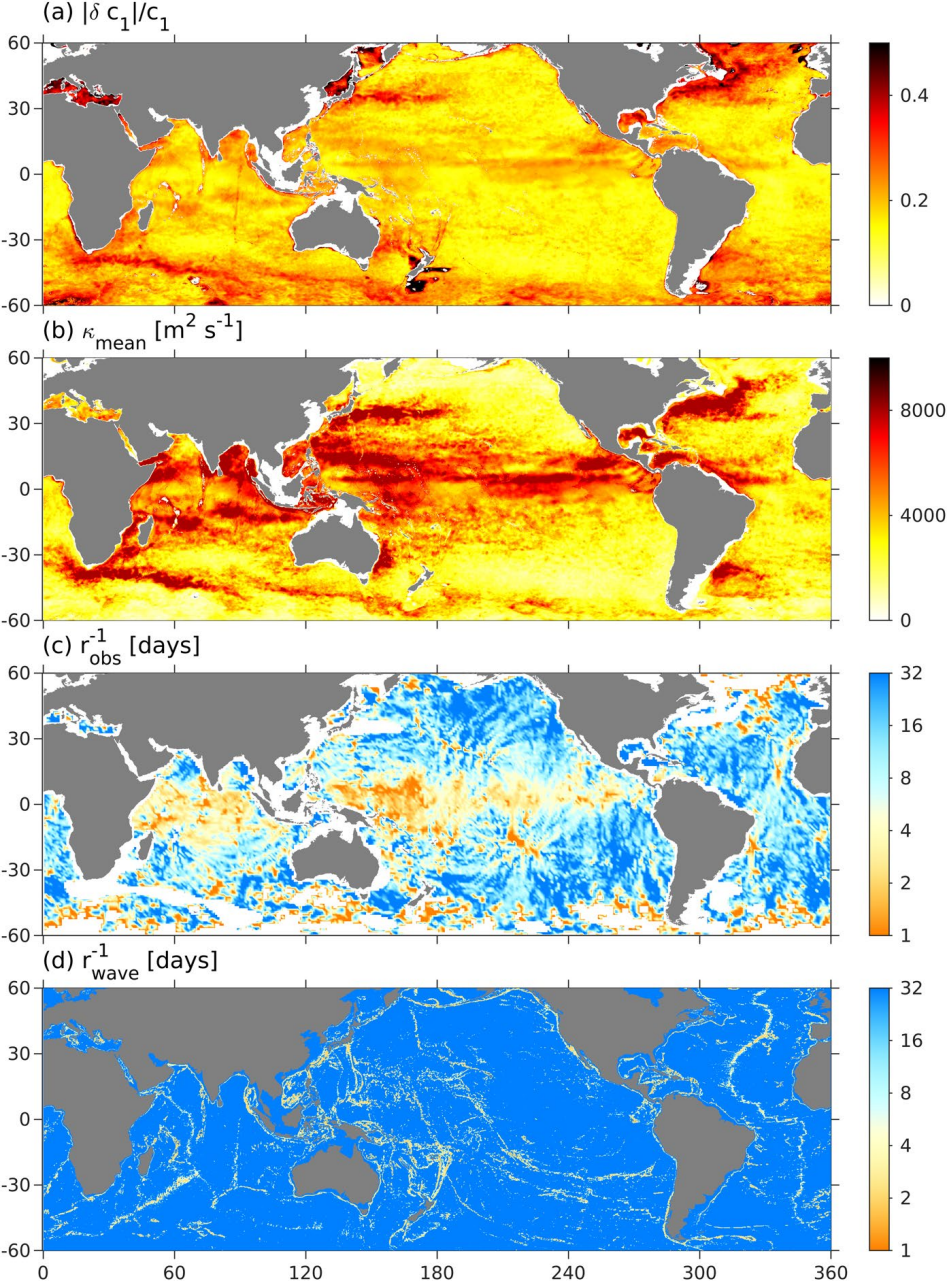
Eigenspeed variability, |*δc*|/*c*, estimated from daily HYCOM snapshots during 2015 (a), the diffusivity of the stationary tide, *κ*_mean_, due to wave‐mean interactions (Savage et al., 2020) (b), the inferred decay time scale, $r_{\text{obs}}^{-1}$ (c), and the wave‐drag decay time scale, $r_{\text{wave}}^{-1}$ (Jayne & St. Laurent, 2001).

A control simulation was run at 1/25° horizontal resolution with 4 vertical modes. The simulation used satellite‐derived bathymetry (Smith & Sandwell, 1997), World Ocean Atlas (WOA) stratification (Locarnini et al., 2010; Antonov et al., 2010), and TPXO8 M_2_ surface tides (Egbert, 1997). The simulation also employed wave drag (Jayne & St. Laurent, 2001)

(7) $$r_{\text{wave}}=\gamma_{r}\frac{1}{2}N_{b}h^{2}/H$$

and diffusion due to a turbulent mean flow (Savage et al., 2020)

(8) $$\kappa_{\text{mean}}=\gamma_{\kappa }\text{Var}\left[\delta c_{1}\right]/\left(2l/c_{1}\right),$$

where *γ*_*r*_ and *γ*_*κ*_ are free parameters, *N*_*b*_ is the buoyancy frequency at the bottom, *h* ^2^ is the bottom roughness (see Jayne & St. Laurent, 2001, for details), *δc*_1_ is the mean eigenspeed perturbation due to mesoscale variability (in HYCOM during 2015) and *l* is an estimate of eddy diameter (see Savage et al., 2020, for details). We set *γ*_*r*_ = 2*π*/(10 km) following Jayne and St. Laurent (2001), and *γ*_*κ*_ = 0.25 to maximize agreement with satellite observations in the control run (Savage et al. 2020) used *γ*_*κ*_ = 1 in the Tasman Sea). Globally, wave drag is negligible (i.e., it has a decay timescale greater than 32 days) except at abrupt topography (mid‐ocean ridges and continental margins), where it has a decay timescale of 1–2 days (Figure 1d). Mean‐flow diffusivity is largest near the equator and in western boundary currents, where it reaches about 5000 m^2^ s^−1^ (Figure 1b). Diffusivity is enhanced in western boundary currents because the fractional variability in eigenspeed, *δc*_1_/*c*_1_, is large (Figure 1a) and enhanced at the equator because eddy diameters are large (see Klocker & Abernathey, 2014).

**Figure 2:**
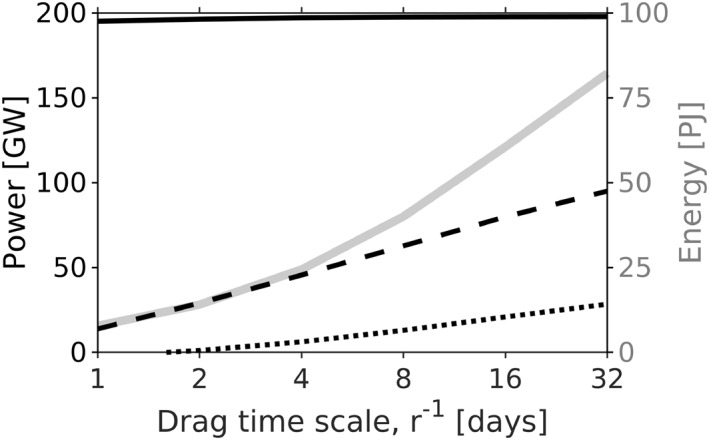
Total simulated mode‐1 generation (*C*_1_; solid), scattering to higher modes (*S*_1_; dashed), flux divergence (onshore flux at the 700 m isobath) (**∇ ⋅F**_**1**_; dotted), and energy (*E*_1_; gray, right axes) as a function of the drag time scale, *r* ^−1^. The left (right) corresponds to high (low) drag simulations. Integrals are preformed below 700 m and the horizontal axes is   log _2_. Positive flux divergence indicates that coastal regions are a sink of mode‐1 energy. Global energy balance residuals (errors) are less than 5 GW.

In addition to the control run, 14 additional simulations were conducted (Figure 2 and Table 1) to examine the effects of using: *r* = *r*_const._ (6 simulations), only *r*_wave_ or *κ*_mean_, HYCOM stratification (averaged from 1994 to 2015; Chassignet et al., 2009), General Bathymetric Chart of the Oceans (GEBCO) topography, FES surface tides (Lyard et al., 2006), 8 vertical modes, and 1/50° and 1/100° horizontal resolution.

**Table 1:** Summary of CSW Simulations

	*C*_1_ [GW]	*S*_1_ [%]	**∇ ⋅F**_**1**_ [%]	*D*_1_ [%]	*R* ^2^
Control	192	16	0	83	0.84
*κ*_mean_ only	194	41	1	55	0.79
*r*_wave_ only	195	21	5	72	0.75
HYCOM strat.	200	17	−1	83	0.84
GEBCO bathy.	225	17	1	81	0.84
FES tides	175	17	5	75	0.79
8 modes	190	17	−1	83	0.83
1/50° (*r*_const._)	232	14	11	75	0.80
1/100° (*r*_const._)	249	9	13	78	0.77

*Note*. Mode‐1 generation (*C*_1_), scattering to Higher Modes (*S*_1_), flux divergence (**∇ ⋅F**_**1**_), and dissipation (*D*_1_) are defined in equation (5). Here, scattering, flux‐divergence, and dissipation are divided by mode‐1 generation to emphasize their relative importance. *R* ^2^ is the correlation between model and satellite SSH amplitude (see Section 2.2). Details of the numerical calculations are provided in Section 2.2. The simulations with different horizontal resolutions employ only $r_{\text{const.}}^{-1}=4$ days.

All simulations are averaged over the 30th or 50th tidal cycle, depending on when the simulation reaches steady‐state (simulations with weaker damping require longer spin‐up). Energy terms in equation (5) are globally integrated below 700 m to (a) omit shallow coastal regions, where surface tides, bathymetry, stratification, and dynamics may be in error and (b) allow a direct comparison with HYCOM (Buijsman et al., 2020).

Simulated SSH amplitudes are compared with the satellite‐derived High Resolution Emperical Tide (HRET) internal‐tide atlas (Zaron, 2019) on a 1° × 1° grid, with each point representing a local average weighted by the HRET mask, which excludes regions where satellite observations are contaminated by coasts or an energetic mesoscale. The fraction of explained amplitude variance is defined *R* ^2^ = 1 − |*A*_obs_ − *A*_sim_|^2^/|*A*_obs_|^2^, where *A* is the mode‐1 SSH amplitude (i.e., a positive real number) averaged over a 1° × 1° area. (Note that |*A*|^2^ is not a true variance because the mean is not removed.) *R* ^2^ ≈ 0.8 because the simulations largely reproduce observed basin‐scale patterns. However, the simulations do not predict the observed phase, so comparisons of complex amplitudes (which include phase information) on the native 1/20° HRET grid yield *R* ^2^ ≈ 0. In other words, the simulated and observed SSH aren't statistically correlated because their phase difference is essentially random. For comparison, HYCOM has a global *R* ^2^ ≈ 0.2 (when predicting amplitude and phase) because it explains about 1/5 the variance of the empirical models (see Table 4 in Carrere et al., 2021), which likely have *R* ^2^ ≈ 1 for stationary mode‐1 variance. In contrast, purely hydrodynamic models are capable of predicting surface tides with *R* ^2^ ≈ 0.9 (e.g., Li & von Storch, 2019).

## 3 Results

Mode‐1 internal‐tide generation is about 200 GW in the CSW simulations, regardless of drag, stratification, bathymetry, and surface tides (Table 1 and Figure 2). GEBCO topography produced slightly larger generation than that of Smith and Sandwell (1997) (225 vs. 192 GW) because it appears to be rougher in some places (we did not attempt to quantify this impression). FES surface tides slightly decreased generation relative to TPXO8.0 surface tides (175 vs. 192 GW) because of slight differences near abrupt topography, where both solutions are uncertain (Stammer et al., 2014). Increased horizontal resolution (from 1/25° to 1/100°) enhanced mode‐1 internal‐tide generation by 26% (from 192 to 249 GW). Increased generation at finer resolution is usually explained by increased high‐mode generation over small‐scale topography (Niwa & Hibiya, 2011, 2014). In CSW we find increased mode‐1 generation at tall, steep topography, which may appear slightly taller and steeper at increased resolution (Di Lorenzo et al., 2006). Mode‐1 generation in HYCOM also increases with resolution (Buijsman et al., 2020). The increase in mode‐1 generation between 1/50° to 1/100° is consistent with *Niwa and Hibiya* (2014)'s global exponential convergence law for total generation with *λ*_CNV_ = 6 deg^−1^ (see their formula).

The control simulation had 192 GW of mode‐1 generation below 700 m. For comparison, Buijsman et al. (2020) reported 220 GW in 1/25° HYCOM and Falahat et al. (2014) reported about 200 GW using weak‐topography theory (see their Figure 9a). Integrated mode‐1 generation versus depth in CSW (not shown) is enhanced in shallow regions and functionally identical to that of semianalytical theory (see Figure 9a in Falahat et al., 2014). Thus, global integrals are sensitive to the minimum cutoff depth. In CSW, 262 GW of mode‐1 generation occur below 250 m, meaning roughly 27% of global generation occurs in the depth range 250–700 m. Buijsman et al. (2020) computed 31% in HYCOM.

Topographic scattering from mode‐1 to modes 2–4 is 30 GW below 700 m and 56 GW below 250 m in the control simulation, consistent with Buijsman et al. (2020)'s calculation of 51 GW below 250 m in HYCOM. In CSW, scattering is largely insensitive to the choices of bathymetry, stratification, and forcing (Table 1). Doubling the number of vertical modes from 4 to 8 only slightly increases scattering at 1/25° resolution, suggesting that only modes 1–4 are resolved at this resolution (see Buijsman et al., 2020). Scattering saturates within about 10 tidal cycles of model spinup (not shown), suggesting that much scattering occurs near generation regions rather than on distant topographic features. A mode‐1 wave generated at a shelfbreak can scatter to higher modes simply by propagating into deeper water (i.e., the vertical wavenumber is constant, but the modal wavenumbers decrease with depth). Local scattering is apparent in high‐drag simulations, where scattering remains nonzero even when waves are unable to propagate farther than a few hundred kilometers (Figure 2). Conversely, increased scattering in low‐drag simulations (including the *κ*_mean_ only simulation) likely occurs at distant topography (Figure 2). The decrease in scattering from the 1/25° to 1/100° simulation is associated with a shift in scattering to water shallower than 700 m. When using a cutoff depth of 100 m, the control and 1/100° simulations have 21% and 20% scattering, respectively. A possible explanation for the reduction in scattering depth is that coastal regions with low resolution artificially reflect onshore propagating waves, enhancing topographic interactions in deeper water.

Flux divergence increases from 0 to 25 GW as drag decreases, meaning that continental margins (shallower than 700 m) increasingly behave as a sink for mode‐1 internal‐tide energy (Figure 2).

Re‐arranging equation (5) with *ν* ≈ 0 and *κ* ≈ 0 produces

(9) $$E_{1}=\frac{C_{1}-S_{1}-\nabla \cdot F_{1}}{2r}$$

for the mode‐1 internal tide. The simulations here indicate that the numerator is roughly constant, so that *E*_1_ and *r* are approximately inversely proportional. Thus, the drag coefficient determines the global energy level, and vice versa. Simulated energy levels are only as accurate as the drag parameterization.

The observed internal‐tide energy implies a corresponding drag coefficient (Figure 2). We estimate the “observed” drag coefficient by interpolating the curve *r*(*A*_sim_) onto the observed SSH amplitude *A*_obs_ on a 1° × 1° grid. This drag, which represents generic unresolved decay processes (Niwa & Hibiya, 2011), has a timescale of 1–4 days near the equator and 16–32 days in the open ocean at mid latitudes (Figure 1c), roughly consistent with respective regions of high and low mesoscale diffusivity (Figure 1b). Note that this decay scale reflects the dynamics of the internal tides as they propagate to a given location, not necessarily the dynamics at that location. The globally averaged decay timescale is 4.5 days, roughly consistent with a 25 PJ stationary mode‐1 tide that loses 50 GW though topographic scattering and <10 GW to dissipation at the continental margins (Figure 2).

**Figure 3:**
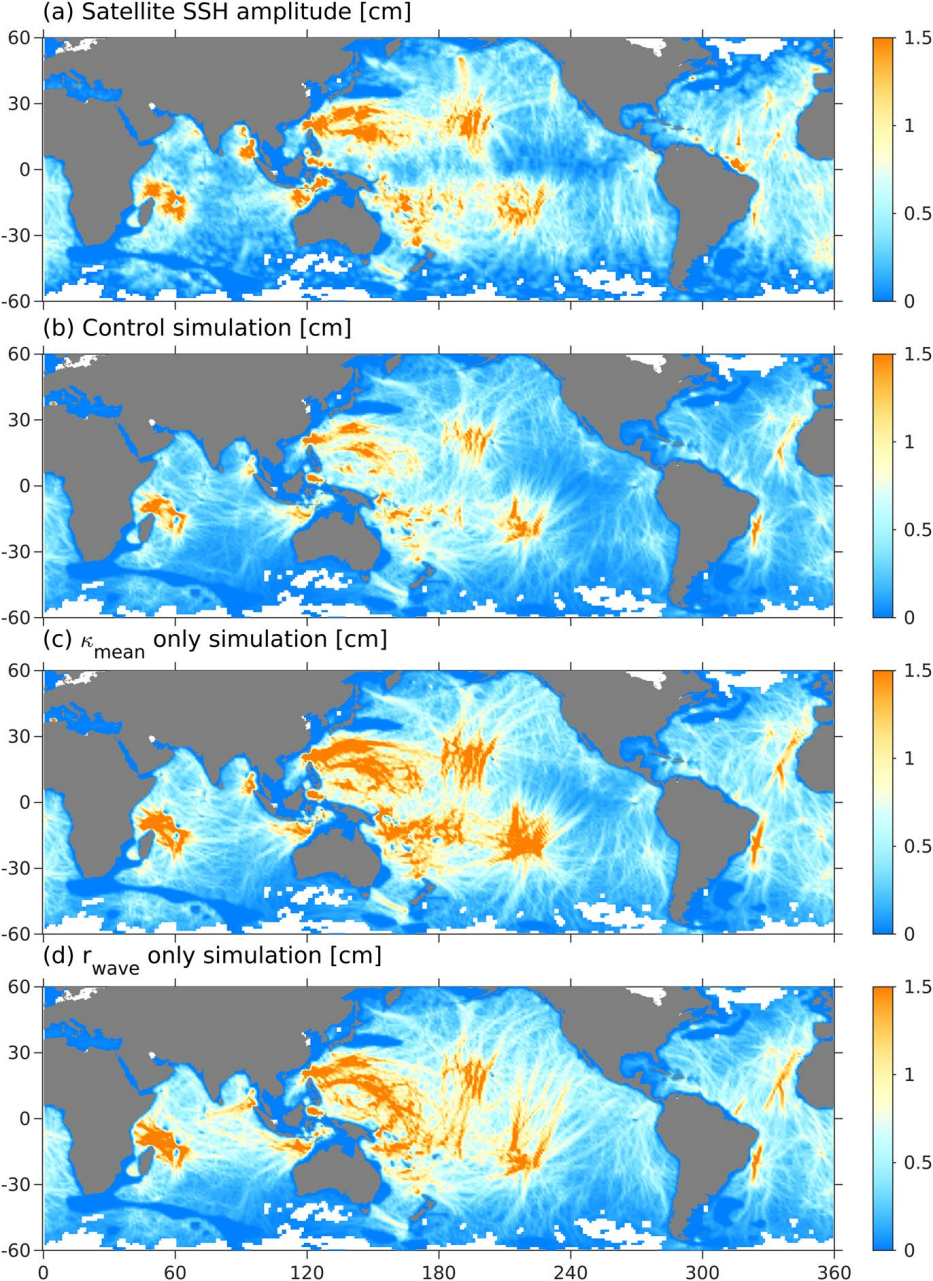
Satellite SSH amplitude (a), and simulated SSH amplitudes from the control simulation (b), *κ*_mean_ only simulation (c), and *r*_wave_ only simulation (d).

The control simulation, which employs wave drag and mesoscale diffusivity, maximizes agreement with observations, yielding *R* ^2^ = 0.84 (Table 1 and Figures 3a and 3b). In contrast, the simulation with *κ*_mean_ only is too energetic at mid‐ocean ridges, but accurately reproduces low energy regions in the eastern equatorial Pacific (Figure 3c), while the simulation with *r*_wave_ only has more accurate energy levels at mid‐ocean ridges, but is too energetic in the eastern equatorial Pacific (Figure 3d). Together, wave drag and mean‐flow diffusion largely explain the spatial distribution of observed mode‐1 SSH amplitude. Since we did not apply a wave‐wave interaction parameterization, wave drag and mean‐flow diffusion may have compensated for that omission. I.e., nonlinear wave‐wave interactions are expected to be large near tall, steep generation sites where energy is large (Sun & Pinkel, 2013; de Lavergne et al., 2019), but these regions also have large wave drag.

## 4 Conclusions

CSW mode‐1 internal‐tide generation agrees with HYCOM and semianalytical theory, regardless of the chosen dissipation parameterizations, stratification, bathymetry, and surface‐tide forcing. All three prediction methods appear to include the relevant dynamics for internal‐tide generation in the deep ocean. Generation dynamics in shallow water are less certain; all three methods show enhanced generation in shallow coastal regions, where wavelengths are short, tidal currents are large, and topography is steep. In shallow regions, CSW has the advantage of high horizontal resolution (e.g., 1/100°), while HYCOM has the advantage of negative feedback between stratification and internal‐tide generation (e.g., overly energetic internal tides can mix the coastal ocean reducing internal‐tide generation). Work is needed to verify global estimates of internal‐tide generation in shallow water.

Model estimates of internal‐tide energy are only as accurate as their drag/dissipation parameterizations (Figure 2). Observations of stationary mode‐1 SSH amplitude are largely reproduced in CSW using simple parameterizations, with minimal tuning, based on wave drag (equation (7)) and mean‐flow diffusion (equation (8)). The wave‐drag parameterization is well established (e.g., Jayne & St. Laurent, 2001; Buijsman et al., 2016, 2020) and the mean‐flow diffusion parameterization is based on large‐scale wave‐mean interactions (Savage et al., 2020) that are resolved in the present generation of global circulation models (e.g., Buijsman et al., 2017; Nelson et al., 2019). We hypothesize that any model with these prerequisites will produce reasonable estimates of the stationary mode‐1 internal tide.

The results here indicate that equation (1) adequately describes the leading‐order dynamics of the stationary internal tide. The numerical implementation (CSW) is computationally efficient without making the weak‐topography approximation, and avoids numerous complexities associated with running a general circulation model. The linearized system is useful because SSH solutions have a simple dependence on the model inputs and parameters (i.e., stratification, surface tides, topography, and drag coefficients), which might be tuned to generate phase‐resolving predictions of low‐mode internal tides. The simplified system is also suitable for inverse modeling.

**Figure 4:**
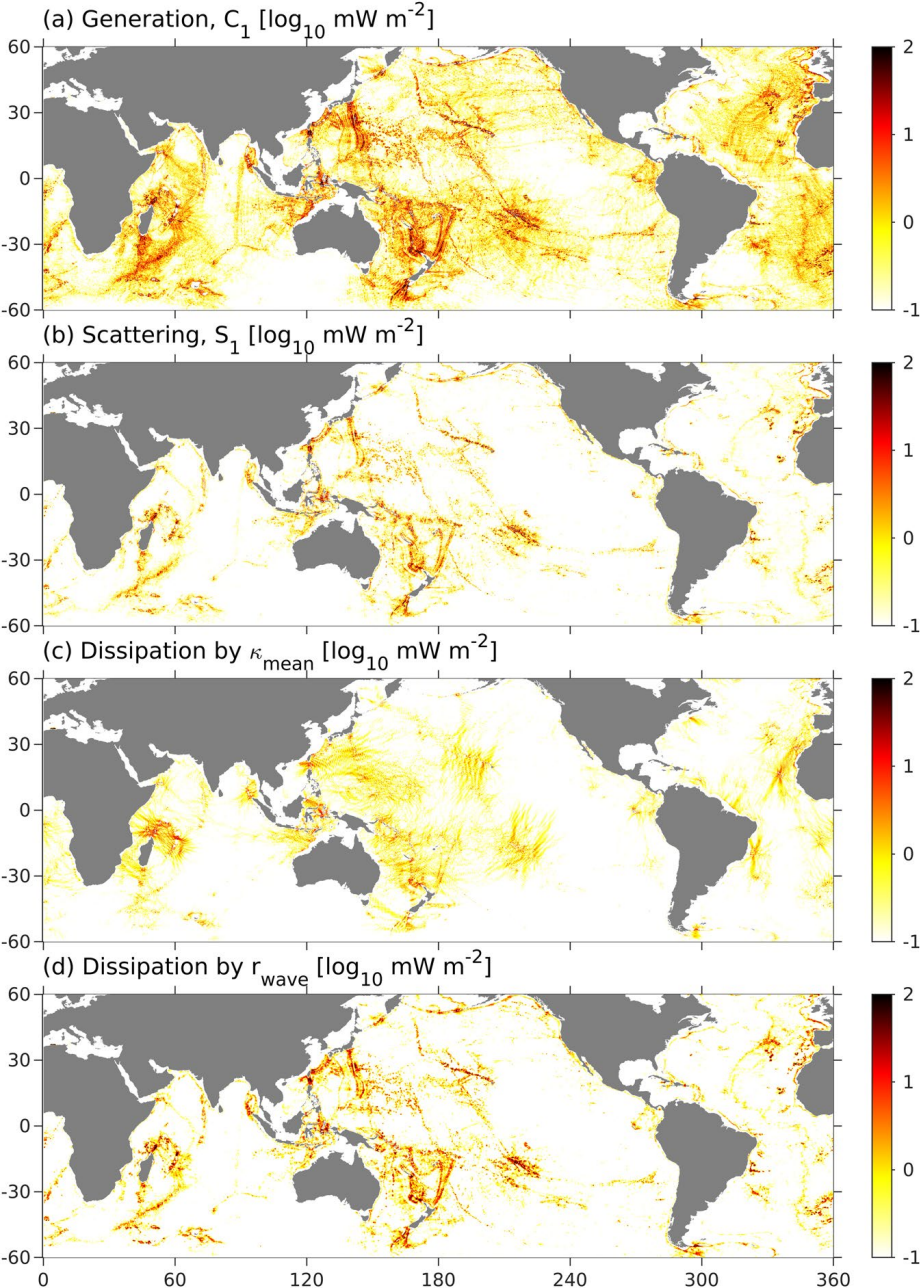
Generation, *C*_1_ (a), scattering (to modes 2–4), *S*_1_ (b), and decay by mean‐flow diffusion (c) and wave drag (d).

The broad agreement between internal‐tide amplitudes in the control run and satellite altimetry (Figure 3), suggest that the simulated energy balance is approximately correct. The simulation indicates about 200 GW of mode‐1 internal tide generation (Figure 4a), which is primarily balanced by scattering to low modes (modes 2%–4%, 16%, Figure 4b) and higher modes (as parameterized by wave drag, 54% Figure 4d). About half of the low‐mode scattering is local to the generation region, while the other half occurs at distant topography (Section 3). The remaining power (29%) is transferred to nonstationary internal tides though mean‐flow diffusion (Figure 4c). Less than 5% of internal‐tide energy is lost by transmission across the 700‐m isobath to shallower water (Table 1). The exact contributions of wave drag and mean‐flow diffusion could vary slightly if the parameterizations were reformulated or re‐tuned, or if a parameterization for wave‐wave interactions was added.

The substantial transfer of energy to the nonstationary mode‐1 tide presents a major challenge for predicting internal‐tide SSH and mapping internal‐tide driven mixing. The time‐dependent generation of nonstationary mode‐1 tides might be predicted by a model with large‐scale wave‐mean interactions, but accurate models of the nonstationary tide require either global observations of nonstationary energy to constrain dissipation parameterizations, or highly accurate dissipation parameterizations to constrain nonstationary energy. Unfortunately, long‐term observations of nonstationary tides in the deep ocean are rare (with the exception of Zaron, 2017) and more accurate parameterizations will likely need to account for complicated small‐scale processes that were ignored here, such as wave‐wave interactions and wave‐mean energy exchanges (e.g., Pollman et al., 2017).

## Data Availability

Simulations were conducted at the Minnesota Supercomputing Institute. The CSW source code is documented in Savage et al. (2020). Data, CSW configuration files, and plotting scripts are available at the Data Repository of the University of Minnesota (DRUM; https://doi.org/10.13020/cf80-eh04).
